# Health Education Resources Favored by Out-Patients Attending King Saud Medical City, Riyadh

**DOI:** 10.7759/cureus.67089

**Published:** 2024-08-17

**Authors:** Abdulrahman M Elnasieh, Atheer T Alturki, Razan Alhadlaq, Mohammed Almesned, Akram N Al-Hazm, Hareth Almajid, Taghered Khalid Khalaf Alharbi, Taghreed M Alaidarous, Aseel T Alturki

**Affiliations:** 1 Family Medicine, King Saud Medical City, Riyadh, SAU; 2 Community and Family Medicine, King Saud Medical City, Riyadh, SAU; 3 Physiotherapy, Dr. Suliman Al Habib Medical Group, Riyadh, SAU

**Keywords:** patient education material, health literacy, text-based information, audio-visual information, audio information

## Abstract

Background

Health education, primarily through printed materials, is crucial for promoting preventive healthcare. It is essential to understand patients' preferences and ensure patient engagement in healthcare decisions. Health literacy challenges persist, and web-based platforms are expanding access. Tailoring materials to target populations, considering content, layout, and cultural appropriateness, maximizes effectiveness. This study aimed to determine the preferred health education resources for patients visiting King Saud Medical City, Riyadh.

Methodology

A cross-sectional study was conducted at King Saud Medical City, Riyadh, targeting adults in outpatient and staff clinic waiting areas. Data were collected using a validated questionnaire and analyzed with IBM SPSS Statistics for Windows, Version 29.0 (Released 2023; IBM Corp., Armonk, New York, United States).

Results

The study included 210 participants and revealed key preferences in health education. Notably, 57.6% preferred social media platforms, while 49.1% favored a doctor as their primary source. Participants prioritized pictures/photos (27.8%) and labels (56.2%) for printed materials. Most participants (91.4%) chose formats based on the comprehensive content provided. Participants sought general health guidance (26%) and preferences were significantly associated with education levels (p=0.010) and different sources (p<0.001). Additionally, students showed a significant association with receiving health education (p=0.046).

Conclusion

The current study revealed diverse preferences for health education resources among patients at King Saud Medical City. The most favored method was social media platforms (57.6%, n=121), with participants ranking it as their first preference. Understanding these preferences is crucial for tailored and effective health education strategies.

## Introduction

Gaining insight into patients' utilization patterns and their preferences regarding printed materials can enhance the dissemination of health information, subsequently leading to an augmentation in healthcare quality [1,2].

Presently, health education has been acknowledged as a pivotal factor in attaining enhanced healthcare results. Elevated patient contentment with educational services is currently correlated with enhancements in healthcare quality [3]. A European health literacy survey found that all adults in the eight included European countries have inadequate health literacy [4]. Health-related information and patient education are now expanding beyond traditional print and oral methods. They are increasingly being delivered through web-based platforms and email communications. This shift is making information and guidance on health maintenance, disease management, and overall well-being more readily available to a broader and more diverse audience [5]. However, it is worth noting that making adjustments to the layout and format can have a substantial impact on enhancing comprehension and the ability of patients to find information within information leaflets [6].

Health education, primarily conveyed through printed materials, plays a pivotal role in promoting preventive healthcare. Giguère et al. showed that printed materials enhance healthcare professionals' practice and patient outcomes as widely employed dissemination strategies [7]. Enhancing patient engagement and addressing health literacy challenges are vital. Web-based platforms extend information access; Tonsaker et al. showed that online health information can increase patients' knowledge of, competence with, and engagement in health decision-making strategies [8]. Beyond readability, format, and layout impact the comprehension of patients [9]. Tailoring materials to the target population ensures effectiveness in promoting positive health behaviors.

Hence, this study was conducted to (i) delineate the patient education materials preferred by the participants, (ii) evaluate the impact of current technology on the selected approach to patient education, (iii) empower study patients by offering them the chance to share their insights on their preferred approach for the provision of health education and services, and (iv) develop a modern directive about the delivery of these essential services.

This study aimed to understand out-patient preferences regarding health education resources in King Saud Medical City, Riyadh, and how these preferences were influenced by various demographic factors. The findings provide valuable insights into the diverse needs and expectations of patients, shedding light on potential improvements in health education delivery.

## Materials and methods

This was a cross-sectional study conducted in the outpatient and staff clinic waiting areas of King Saud Medical City (KSMC), a major tertiary care center in Riyadh, Saudi Arabia, with 1,500 beds and over one million outpatient visits annually. The study was conducted between November 1, 2023, and December 31, 2023. The study was approved by the Institutional Review Board of King Saud Medical City of Research and Innovation Center (approval number H1RI-02-Oct23-02, dated October 10, 2023). The researchers guaranteed that respondents' identities remained anonymous, and the collected data remained confidential. Participants who opted to take part in the study signified their agreement by selecting the "agree" box, thus providing informed consent for the data collection process. An Arabic-translated consent form was available for all the participants to view for signing.

Inclusion and exclusion criteria

This study was conducted based on a convenience sampling technique. Hence, all adult patients attending the study facility during the study period and who were willing to participate in the study after counseling were included. Patients visiting the laboratory, radiology department, dental clinics, and physical therapy department along with pediatrics and obstetrics and gynecology patients during the research timeframe were excluded from the study. Additionally, the study did not involve the participation of the relatives accompanying these patients.

Data collection tool

A self-administered, pre-validated, cross-sectional, and close-ended structured survey was used in this study after certain modifications were adopted from different studies in the field [1,4]. The questionnaire was translated into Arabic for easy understanding of the participants and then again to English after the responses were obtained.

Study variables

In this research endeavor, the focus was on the dependent variable, which revolved around the health education resources preferred by patients. The independent variables encompassed various elements, such as the participants' demographic and personal traits. This scope also extended to investigating the specific health education materials the participants favored for delivery and delving into the rationale behind their selections.

Statistical analysis

The statistical analysis was conducted encompassing both descriptive and inferential methodologies. Firstly, a descriptive analysis was conducted to summarize the demographic characteristics of the participants, which include age, gender, and other features. This provided an overview of the study population after which the Shapiro-Wilk test was conducted to assess the normality of the data. Subsequently, Fisher’s exact test and Independent sample T-tests were employed to examine the association of reception of health education with different features. Statistical significance was established at a p-value of 0.05 or lower and a 95% confidence interval (CI). All statistical analyses were executed using IBM SPSS Statistics for Windows, Version 29.0 (Released 2023; IBM Corp., Armonk, New York, United States).

## Results

This study included 210 individuals. The majority were males (64.8%, n=136), with a mean age of 37.2 years (SD=11.9). Saudi nationals constituted 90.5% (n=190) of the participants. Marital status varied, with 71.9% (n=151) being married. Educational levels included university graduates (57.6%, n=121). Regarding occupation, diverse categories were observed, including medical professionals (29.5%, n=62), employees (17.1%, n=36), and housewives (8.1%, n=17). The majority of participants received health education materials from the hospital (71.4%, n=150) (Table 1).

**Table 1:** Sociodemographic and other parameters of participants (N=210) The data is presented in frequency (n) and percentage (%), except for age, which was in mean±SD and range. OPD: out-patient department

Parameters		Frequency (Percentage)
Gender	Female	74 (35.2)
	Male	136 (64.8)
Age	Mean±SD	37.2 (11.9)
	Range	4-85
Nationality	Non-Saudi	20 (9.5)
	Saudi	190 (90.5)
Marital Status	Single	55 (26.2)
	Married	151 (71.9)
	Divorced/Widowed	4 (1.9)
Education	No Formal Education	8 (3.8)
	Primary/Intermediate	17 (81)
	Secondary	26 (12.4)
	University	121 (57.6)
	Post-Graduate	38 (18.1)
Occupation	Medical Professionals (Doctors/Nurses)	62 (29.5)
	Employee	36 (17.1)
	Housewife	17 (8.1)
	Administrative	17 (8.1)
	Teacher	17 (8.1)
	Student	15 (7.1)
	Unemployed	13 (6.2)
	Retired	10 (4.8)
	Other	23 (11)
Sample Source	Staff Clinic	49 (23.3)
	OPD	161 (76.7)
Recieved Any Health Education Materials or Advice from this Hospital?	No	60 (28.6)
	Yes	150 (71.4)

**Figure 1:**
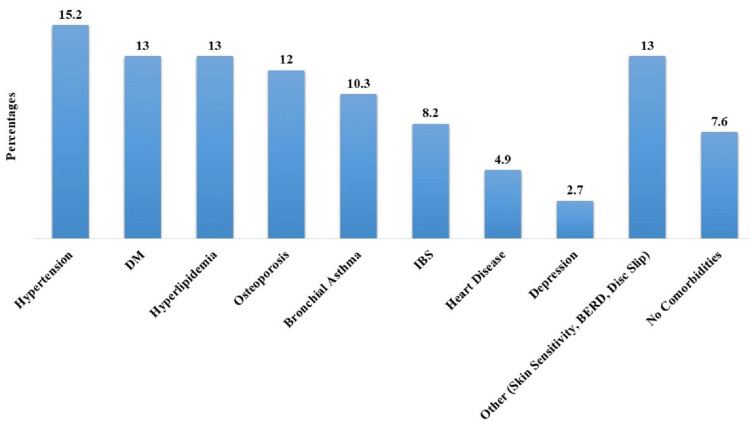
Prevalent co-morbidities among the participants (N=210) IBS: irritable bowel syndrome; DM: diabetes mellitus

The most commonly reported conditions included hypertension (n=32, 15.2%), diabetes (n=27, 13%), and high fat levels (n=27, 13%). Other prevalent conditions included osteoporosis (n=25, 12%), bronchial asthma (n=22, 10.3%), and other comorbidities such as depression, irritable bowel syndrome (IBS), heart disease, and others. Approximately 7.6% (n=16) reported no specific health condition (Figure 1).

**Figure 2:**
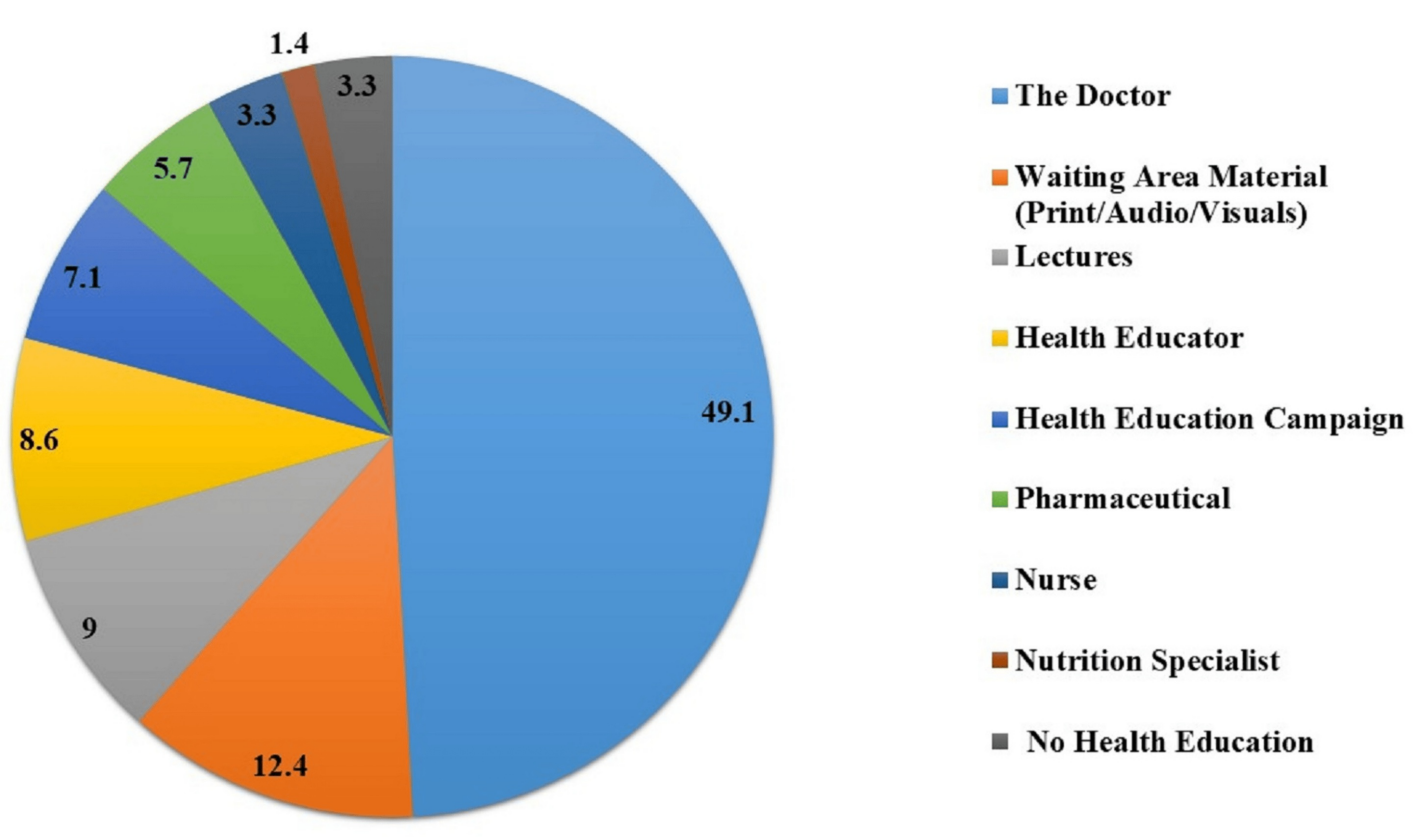
Percentages of different sources of health education for the participants (N=210)

The primary source of health education material or advice was the physician in 49.1% (n=103) of the participants, followed by waiting area material (print/audio/visuals) at 12.4% (n=26). Lectures, health educators, and health education campaigns contributed significantly, with 9% (n=19), 8.6% (n=18), and 7.1% (n=15), respectively. Other sources, including pharmacists, nurses, and nutrition specialists, showed lower percentages, while 3.3% (n=7) reported receiving no health education (Figure 2).

For health education services, the most favored method was social media platforms, with most participants (57.6%, n=121) ranking it as their first preference. Group discussion with education and health education clinics closely followed as the second preference (55.7%, n=117 each). Traditional media (50.0%, n=105), including radio and television, was also popular, along with social media videos and reels (51.0%, n=107). Regarding healthcare professionals for health education, the majority preferred physicians (77.2%, n=162) as their first choice. Nurses (53.8%, n=113) and health educators (57.6%, n=121) were also highly regarded. This indicates a strong inclination towards medical professionals for health education delivery (Table 2).

**Table 2:** Participant preferences for the method and professional for delivery of health education (N=210) The data is presented in frequency (n) and percentage (%).

Methods/Professionals	Participants Response in Order of Preference		
	First preference, n (%)	Second preference, n (%)	Third preference, n (%)
Preferred Method for Health Education Services			
Group Discussion with Education	117 (55.7)	37 (17.6)	56 (26.7)
Health Education Clinics	117 (55.7)	58 (27.6)	35 (16.7)
Traditional Media (Radio and TV)	105 (50.0)	44 (21.0)	61 (29.0)
Social media platforms (Facebook, Twitter, WhatsApp, etc.)	121 (57.6)	60 (28.6)	29 (13.8)
Social media videos and reels	107 (51.0)	52 (24.8)	51 (24.3)
Lectures, Conferences, Seminars, and Workshops	81 (38.6)	58 (27.6)	71 (33.8)
Podcasts	77 (36.7)	57 (27.1)	76 (36.2)
Drama and Plays	73 (34.8)	48 (22.9)	89 (42.4)
Health Education Publications	92 (43.8)	42 (20.0)	76 (36.2)
Preferred Healthcare Professionals for Health Education			
Physician	162 (77.2)	24 (11.4)	24 (11.4)
Nurse	113 (53.8)	53 (25.2)	44 (21.0)
Health Educator	121 (57.6)	49 (23.3)	40 (19.0)
Nutritionist	110 (52.4)	55 (26.2)	45 (21.4)
Pharmacists	97 (46.2)	58 (27.6)	55 (26.2)
Others	69 (32.9)	51 (24.3)	90 (42.9)

**Figure 3:**
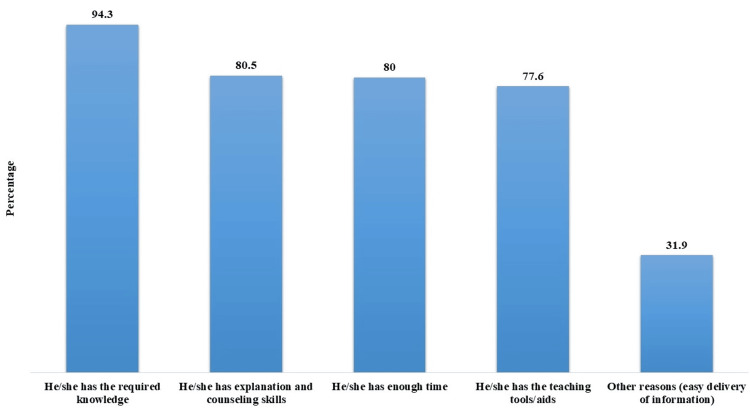
Rationales for first choice preference of the professional delivering health education (N=210)

The majority (94.3%, n=198) cited the professional's possession of required knowledge as the primary reason for their first preference of the method or professional delivering health education. Explanation and counseling skills were crucial for 80.5% (n=169), while 80% (n=168) valued the availability of sufficient time. Teaching tools/aids were important for 77.6% (n=163) of participants. Additionally, 31.9% (n=67) mentioned other reasons, such as the easy delivery of information (Figure 3).

The first preference for receiving printed materials was labels, chosen by 56.2% (n=118) of the participants. Tri-fold brochures (48.1%, n=101) and sample cards (49.0%, n=103) were also popular choices for the first preference. Plain paper (45.7%, n=96) and manuals (39.0%, n=82) received substantial preferences. In the second and third preferences, participants expressed a variety of choices, with responses distributed across different formats (Table 3).

**Table 3:** Preferred format for receiving health education printed materials among participants (N=210) The data is presented in frequency (n) and percentage (%).

Format of education printed material	Participant Response in Order of Preference		
	First preference, n (%)	Second preference, n (%)	Third preference, n (%)
Plain Paper	96 (45.7)	32 (15.2)	82 (39.0)
Tri-Fold Brochure	101 (48.1)	58 (27.6)	51 (24.3)
Manuals	82 (39.0)	55 (26.2)	73 (34.8)
Sample Cards	103 (49.0)	51 (24.3)	56 (26.7)
Labels	118 (56.2)	53 (25.2)	39 (18.6)
Other	61 (29.0)	37 (17.6)	112 (53.3)

**Figure 4:**
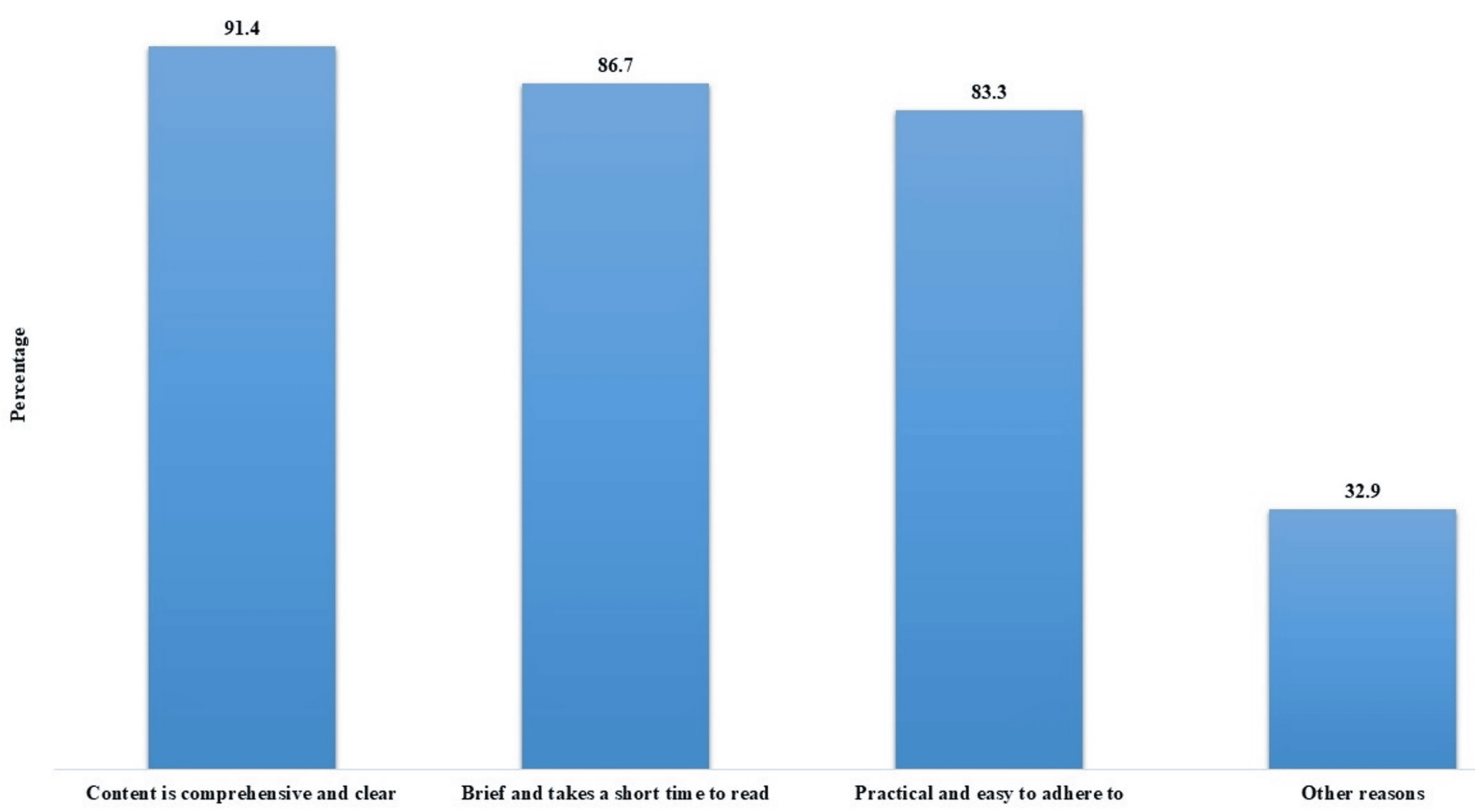
Rationales for first choice preference for health education printed material format (N=210).

The overwhelming majority (91.4%, n=192) prioritized comprehensive and clear content. Additionally, 86.7% (n=182) valued materials that were brief and could be quickly absorbed. Practicality and ease of adherence were crucial for 83.3% (n=175) of participants. A smaller percentage (32.9%, n=69) cited other reasons (Figure 4).

**Figure 5:**
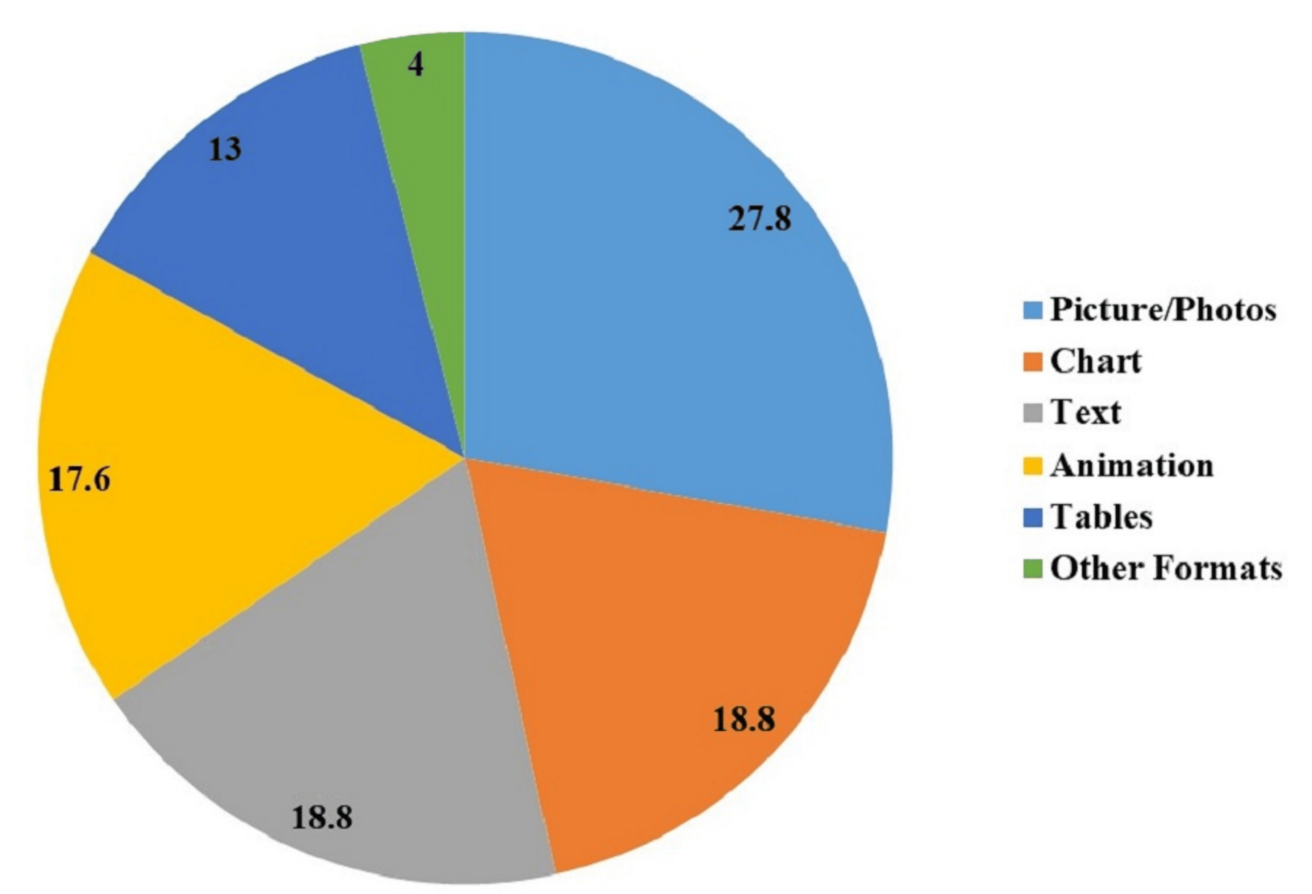
Preference of shape/format for health education printed materials

Over one-quarter of the participants (27.6%, n=58) favored pictures/photos, followed by charts and text (18.7%, n=39 each). Animation was preferred by 17.6% (n=37), while tables were chosen by 13% (n=27). A smaller percentage (4%, n=9) opted for other formats (Figure 5).

**Figure 6:**
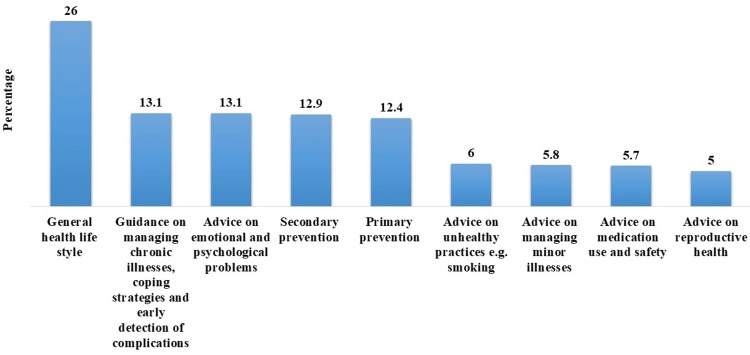
Specific information participants hope to find in the health education materials

Again over one-quarter of the sample (26%, n=55) sought guidance on general health lifestyle. About 13.1% (n=28) were interested in information on managing chronic illnesses, coping strategies, early detection of complications, and advice on emotional and psychological problems. Secondary prevention was important for 12.9% (n=27), while 12.4% (n=26) sought information on primary prevention. A smaller percentage was interested in advice on unhealthy practices (6%, n=13), managing minor illnesses (5.8%, n=12), medication use and safety (5.8%, n=12), and reproductive health advice (5%, n=11) (Figure 6).

Gender, age, and nationality did not exhibit a significant association with receiving health education materials. Occupational status displayed a significant association, particularly with students (93.3%, n=14) who received more education as compared to others (p-value=0.046). Marital status was not significantly associated (p-value=0.127), but a significant association was observed concerning education levels, indicating that individuals with no formal education (87.5%, n=7) and secondary education (88.5%, n=23) were more likely to receive health education (p-value=0.010). Moreover, the source of previous health education significantly influenced participants' likelihood of receiving information. Associations were highly significant for various sources, including health education campaigns, health educators, lecturers, nurses, nutrition specialists, pharmacists, and physicians (p-value<0.001) (Table 4).

**Table 4:** Receipt of health education distributed according to demographic characterisitcs The data is presented in frequency (n) and percentage (%). ^a^Fisher’s Test, ^b^Independent T Test

Demographic Characteristics		Ever received any advice/health education materials from this hospital		P- Value
		No, n (%)	Yes, n (%)	
Gender	Female	19 (25.7)	55 (74.3)	0.526 ^a^
	Male	41 (30.1)	95 (69.9)	
Age	Mean (SD)	38.5 (11.9)	36.6 (11.9)	0.303 ^b^
Nationality	Non-Saudi	2 (10.0)	18 (90.0)	0.068^ a^
	Saudi	58 (30.5)	132 (69.5)	
Occupation	Administrative	3 (17.6)	14 (82.4)	0.046^ a^
	Employee	8 (22.2)	28 (77.9)	
	Housewife	5 (29.4)	12 (70.6)	
	Medical Professionals (Doctors/Nurses)	19 (30.6)	43 (69.4)	
	Retired	6 (60.0)	4 (40.0)	
	Student	1 (6.7)	14 (93.3)	
	Teacher	9 (52.9)	8 (47.1)	
	Unemployed	2 (15.4)	11 (84.6)	
	Other	7 (30.4)	16 (69.6)	
Marital Status	Single	14 (25.5)	41 (74.5)	0.127^ a^
	Married	43 (28.5)	108 (71.5)	
	Divorced/Widowed	3 (75.0)	1 (25.0)	
Education	No Formal Education	1 (12.5)	7 (87.5)	0.010^ a^
	Primary/Intermediate	5 (29.4)	12 (70.6)	
	Secondary	3 (11.5)	23 (88.5)	
	University	32 (26.4)	89 (73.6)	
	Post-Graduate	19 (50.0)	19 (50.0)	
Sample Source	Staff Clinic	15 (30.6)	34 (69.4)	0.721^ a^
	OPD	45 (28.0)	116 (72.0)	
Where Previously Received Health Education	Health Education Campaign	2 (13.3)	13 (86.7)	<0.001^ a^
	Health Educator	6 (33.3)	12 (66.7)	
	Lectures	6 (31.6)	13 (68.4)	
	Nurse	1 (14.3)	6 (85.7)	
	Nutrition Specialist	0 (0.0)	3 (100.0)	
	Pharmaceutical	7 (58.3)	5 (41.7)	
	The Doctor	19 (18.6)	83 (81.4)	
	Waiting Area Material (Print/Audio/Visuals)	11 (42.3)	(57.7)	

## Discussion

The demographic profile of the participants revealed a predominantly male population, with Saudi nationals comprising the majority. This aligns with a previous study by Thompson et al., which indicated variations in healthcare-seeking behaviors across gender [10]. The majority were married and had a relatively high educational background, primarily university graduates, suggesting an educated and family-oriented cohort. The diverse occupational distribution, including a significant proportion of medical professionals, highlighted the importance of tailoring health education resources to meet the specific needs of various occupational groups.

Hypertension, diabetes, and hyperlipidemia emerged as the most commonly reported conditions. These findings correlate with global health trends, emphasizing the importance of addressing lifestyle-related diseases, particularly with printed education material. Bull et al. showed that printed health education materials were widely used to raise awareness, change attitudes, and promote healthy behaviors [11]. Identifying the prevalence of specific health conditions is crucial for tailoring health education materials to address the most pressing concerns of the patient population.

The current study provides insights into the varied sources of health education for participants. The pivotal role of doctors as the primary source highlighted the significance of physician-patient interactions in disseminating health information. Similarly, Brunton et al. showed that physicians have a central role in educating patients and the public about the elements of personal health maintenance [12]. Waiting area material, lectures, health educators, and health education campaigns also contribute significantly. Understanding these sources helps healthcare providers strategically allocate resources and prioritize effective channels for health education delivery.

Moreover, the current study delves into participants' preferences for health education methods and professionals. Social media platforms emerged as a favored method, suggesting the increasing influence of digital platforms in health communication. Similarly, previous studies suggested that the use of social media platforms can positively influence awareness of public health, behavioral changes, and public health protection [13,14]. The preference for group discussions and health education clinics emphasizes the importance of interactive and personalized approaches. Similarly, Yazachew et al. showed that the diverse health education methods range from machine-dependent to interactive (group discussions), emphasizing cost-effectiveness, cultural acceptability, and effectiveness [15]. Doctors remained the preferred healthcare professionals for health education, aligning with the trust and credibility traditionally associated with physicians. Nurses and health educators also play vital roles, indicating a multidisciplinary approach to health education delivery [16]. Notably, healthcare professionals' expertise, effective communication, time availability, and access to teaching tools are crucial aspects of patient preferences, highlighting the need for comprehensive training and resource allocation.

Moreover, the current study explored participants' preferences for the format of health education printed materials. Labels, tri-fold brochures, and sample cards emerged as popular choices, with participants valuing visually engaging and informative formats. The diverse preferences highlight the need for offering a variety of materials to cater to individual learning styles. Similarly, Krontoft et al. showed that patients highly preferred text-based education materials (86.46%), but significant interest exists in video (50.21%) and podcast (31.67%) formats [4]. Moreover, the overwhelming patient’s emphasis on comprehensive and clear content, along with practicality and ease of adherence, reflects a desire for accessible and actionable information. These findings align with health literacy principles, emphasizing the importance of clear and concise communication in health education materials [17].

Participants prefer visually oriented and information-rich formats like pictures/photos, charts, and text for health education materials. Addressing these preferences is crucial for resonating with the audience. Insights revealed a priority for general health lifestyle guidance, followed by managing chronic illnesses, coping strategies, and emotional well-being. Emphasizing not only physical but also mental and emotional aspects is vital in health education materials according to the interest of the patients. Similarly, Craven et al. showed that patients were interested in having access to printed materials about mental health problems [18].

The current study also explored associations between the receipt of health education and participant characteristics. Significant associations were observed for educational levels, occupational status, and sources of previous health education. The finding showed that those with lower education levels were more likely to receive health education emphasizing the need for targeted outreach and tailored materials for diverse educational backgrounds. Additionally, the association with occupational status, especially students, suggested opportunities for enhanced health education in educational institutions.

This study's limitations included potential biases in participant self-reporting, limited generalizability to other populations, and reliance on preferences rather than actual behaviors. Additionally, the study might not have captured evolving preferences in a dynamic healthcare landscape. Preferences expressed in a survey might not translate into actual behaviors due to various external constraints or changes in circumstances. Healthcare environments and patient preferences can change quickly due to new medical technologies, treatments, policies, or changes in societal attitudes. The study’s findings might become outdated if these factors evolve. Without longitudinal data, it is challenging to determine if the preferences captured in the study are stable over time or if they fluctuate with changing healthcare landscapes and personal circumstances.

Further research on health education formats, cultural nuances, and personalized interventions could improve health outcomes. Technology-driven, interactive strategies aligned with cultural preferences can enhance engagement. Continuous updates and adaptations to healthcare strategies are crucial. The study's findings could guide the development of patient-centered, culturally sensitive health education programs.

## Conclusions

This study provides valuable insights into patient preferences for health education resources at King Saud Medical City, Riyadh, where 57.6% preferred social media platforms as a health education source, 27.8% prioritized pictures/photos as printed material, 91.4% emphasized comprehensive content, and 26% of participants wanted guidance on general health. Significant associations of health education were found with the educational levels of the participants (p-value=0.010), and varied sources of health education (p-value<0.001). The diverse preferences highlight the need for personalized health education strategies. Understanding the demographic variations can help in designing more effective educational materials.

The shift towards social media and digital formats indicates a need to integrate these platforms into health education efforts. Implementing these insights can lead to better-informed patients and improved health outcomes. It is recommended to encourage doctors to provide comprehensive health education during consultations and train healthcare professionals on effective communication strategies to enhance patient understanding. Printed materials should be designed with clear labels and visual aids to enhance comprehension. Educational campaigns should on general health topics, as this is a significant area of interest for patients. Targeted health education programs should be developed for students, considering their significant interest and association with receiving health education. Implementing these recommendations can help healthcare providers at King Saud Medical City and similar institutions create more effective and patient-centered health education strategies.
